# A Comparative Study on Bio-Based PU Foam Reinforced with Nanoparticles for EMI-Shielding Applications

**DOI:** 10.3390/polym14163344

**Published:** 2022-08-17

**Authors:** Vinoth Kumar Selvaraj, Jeyanthi Subramanian

**Affiliations:** School of Mechanical Engineering, Vellore Institute of Technology, Chennai 600127, Tamil Nadu, India

**Keywords:** bio-based PU foam, EMI shielding effectiveness, COMSOL Multiphysics, response surface methodology

## Abstract

Today, most commercial polyols used to make polyurethane (PU) foam are produced from petrochemicals. A renewable resource, castor oil (CO), was employed in this study to alleviate concerns about environmental contamination. This study intends to fabricate a bio-based and low-density EMI-defending material for communication, aerospace, electronics, and military appliances. The mechanical stirrer produces the flexible bio-based polyurethane foam and combines it with nanoparticles using absorption and hydrothermal reduction processes. The nanoparticles used in this research are graphite nanoplates (GNP), zirconium oxide (ZrO_2_), and bamboo charcoal (BC). Following fabrication, the samples underwent EMI testing using an EMI test setup with model number N5230A PNA-L. The EMI experimental results were compared with computational simulation using COMSOL Multiphysics 5.4 and an optimization tool using response surface methodology. A statistical design of the experimental approach is used to design and evaluate the experiments systematically. An experimental study reveals that a 0.3 weight percentage of GNP, a 0.3 weight percentage of ZrO_2_, and a 2.5 weight percentage of BC depict a maximum EMI SE of 28.03 dB in the 8–12 GHz frequency band.

## 1. Introduction

Polyurethane (PU) is a polymer that comes under the combination of carbonate links. In the 20th century, polyether polyols were cheaper and easier to handle. Meanwhile, they were more resistant to water compared with polyester polyols. This was the primary reason polyether polyols gained popularity. Polyurethane is conventionally molded by a combination of diisocyanate or tricyanide with polyol under exposure to UV light in the presence of a catalyst. Polyurethanes are thermosetting polymers that withstand high temperatures (120 °C to 300 °C) and have good solvent resistance [1,2,3].

PU foam is receiving specific interest in material science and technology, and it is demarcated as connecting the breach between plastic and rubber materials. Polyether polyols based on polypropylene, polyethylene oxide or tetrahydrofuran, aliphatic polyester polyols, aromatic polyester polyols, polycarbonate polyols, acrylic polyols, and polybutadiene polyols are the most commonly used polyols in the production of polyurethanes. Toluene diisocyanate (TDI) and methylene diphenyl diisocyanate (MDI) are two aromatic isocyanates commonly used in polyurethanes [4]. Aliphatic isocyanates such as isophorone diisocyanate (IPDI) and hexamethylene diisocyanate (HDI) are used in a few applications. Aromatic types are more reactive and are used in various applications, including flexible and rigid foams [4]. Previously, petroleum-based polyols were used to fabricate PU foam. These synthetic polyols tend to be toxic, non-degradable, and high-cost, resulting in many health hazards and environmental pollution. The alternative source for synthetic polyol is a natural one which has eco-friendly, biodegradable nature, low-cost, and unique thermal properties. Therefore, regarding the ecological concern, we substitute synthetic polyol with vegetable oil-based polyol to create an organic foam [5,6,7]. However, various vegetable oils, such as sunflower, castor, and soybean oils, are being proposed to partially or entirely replace these oil-based polyols [8]. To make a flexible and bio-based PU foam, we use a combination of castor oil-based polyol with toluene diisocyanate (TDI) as base ingredients. During the 1960s, PU foam was more attractive in the automobile and aircraft industries, as PU foams are used for safety components such as door panels, etc. It gradually gained prominence in all technology areas and is now used in the automobile industry. Foams are widely used in auditoriums, optical fibers, engine covers, and other applications due to their acoustic, electromagnetic, and thermal properties. The use of polyurethanes and the quantity of their scrap are increasing. This PU foam is recycled in various ways to reduce waste, and it has also been reused for multiple applications [9,10,11]. However, they have many applications in thermal properties; they do not withstand high temperatures and they are an inferior adhesive to metal structures. To overcome these limitations, adding nanoparticle materials such as GNP, ZrO_2_, and BC is a choice using hydrothermal processes, which provide good results due to their physical and chemical properties. Recent research has discovered that when two distinct nanoparticles are used as filler materials, they have synergistic benefits [12,13,14,15].

Recently, exciting facts have been discovered about nanocomposites and they have surprised researchers with their unique properties [16,17], and gained more scope in the field of research and development [18,19]. Graphene nanoparticles have developed a prominent role due to their incomparable mechanical, electrical, and thermal properties. It is recorded as the finest and thinnest 2D material among all materials. It is the most in-demand material in research and development in the application of semiconductors in electric batteries and polymer composites. Over a wide range of applications, graphene is considered to have unique permittivity properties, from microwave to millimeter frequency. Furthermore, it has been found that small capacitors fabricated using graphene can store large amounts of electrical energy. Notably, graphene nanoparticles are good at saturable absorption, which is applied over ultrafast photonics. Despite promising results in different applications, preliminary studies have revealed the complete potential biocompatibility of graphene-based materials [20,21,22].Zirconium oxide nanoparticles mark a prominent place and are emerging in the field of nanotechnology. These nanoparticles are applied in particular applications, as they possess unique properties in electronics, biotechnology, and cosmetics [23].

Generally, zirconium is available in the form of nanofluids and nanoparticles. Zirconium is applied to insulating and fire-retardant materials, as they can resist high temperature up to 2715 °C. Graphene in powder form exhibits optical and pyro-optical properties, which are extensively used in optical storage industries [24,25]. High ionic conductivity makes it one of the most useful in electroceramics [26,27,28]. Bamboo charcoal is produced by a carbonization process; it has excellent properties, such as high conductivity, adsorption properties, and a massive surface area. It is an environmentally functional material with unique absorption properties and has a large surface area with an efficient porous structure, making it more suitable for absorption into PU foam [29,30].

Electromagnetic interference (EMI) or noise pollution happens due to the advancement of using large amounts of electromagnetic (EM) appliances in various industrial applications. Beyond EMI shielding, the essential operation is based on the Lorentz force law; a wave is an incident on the shielding’s surface, and the electrons in the shield interact with the incident EM wave to create an EM field. The power of the input EM wave first reduced the induced EM field. We take three parameters in a complete set of EMI-shielding operations: absorption, reflection, and multiple reflections [31,32,33]. Later, this was difficult to construct because the materials required to build it were more costly. As a result, to achieve the intended results, the appropriate shielding material must be given under specific conditions. It is also critical to consider the performance characteristics of various EMI-shielding materials. In research, many conductive materials were adopted to study EMI shielding effectiveness. Though traditional metals such as aluminum and steel are widely employed for EMI shielding due to their high conductivity, they lack bulk and are prone to corrosion [34,35,36]. Incorporating conductive fillers, such as carbon black, carbon nanotubes (CNT), nano graphite, and graphene, into polymeric matrices is an appropriate method for fabricating conductive polymer nanocomposites as EMI-shielding materials [37,38]. For example, Zhang et al. used CO_2_ foaming to create porous poly(methyl methacrylate)/graphene composite foams with an EMI Shielding Effectiveness (SE) of 13–19 dB and a density of 0.79 g/cm^3^ [39]. Qiuyue Jiang et al. created flexible thermoplastic polyurethane/reduced graphene oxide (RGO) composite foams with a shielding effectiveness of 21.8 dB using a supercritical CO_2_ foaming method and 3.17 vol.% RGO [40]. Yang et al. demonstrated that polystyrene/MWCNT composites with a 7 wt.% MWCNT loading achieved a shielding effectiveness (SE) value of 20 dB [41]. In contrast, Basuli et al. investigated the EMI shielding response of ethylene methyl acrylate/MWCNT composites. They discovered an SE value of 22 dB in the X-band at a 12 wt.% MWCNT loading [42]. A multifunctional composite foam with high EMI, high electrical conductivity, and excellent photo-electro-thermal properties may be valuable in various applications, such as sensing, actuating, energy harvesting, and many other fields [43,44].

COMSOL Multiphysics is a finite element analysis simulation software. It provides a comprehensive solution and a unified, integrated development environment in mechanical, fluid dynamics, electromagnetic interference, acoustics, and chemical applications. COMSOL can be defined as a versatile general simulation analysis software that is mainly based on advanced numerical methods [45]. While comparing with other analysis tools such as Ansys and LS DYNA, COMSOL Multiphysics is a user-friendly tool where we can combine multiple physics simultaneously. In contrast, it is quite challenging and time-consuming to solve a simple dynamic problem with Ansys. More simply, COMSOL Multiphysics is oriented toward academia, whereas Ansys is industry-oriented [46,47].

RSM (Response Surface Methodology) is a statistical technique for DOE (Design of Experiments) that effectively identifies the link between many variables and one or more response variables. The primary goal of RSM in experimental design is to find the best response or output to a given input variable. It is an easy way to evaluate polynomial techniques used as factorial experiments. RSM is essential in the real-time investigation, as both the parameters are unknown. In addition, it is beneficial for improving the experiment’s performance with a smaller number of trials [48,49,50].

In this paper, a novel study is carried out where we use nanoparticles such as GNP, ZrO_2_, and BC simultaneously as reinforcement in flexible bio-based polyurethane foam for electromagnetic defense applications. The impacts of the weight percentage of GNP, ZrO_2_, and BC on EMI shielding effectiveness are evaluated. Further, the RSM, one of the DOE techniques for optimizing the best combination of nanoparticles that yields better shielding effectiveness, is adopted and compared with experimental and simulation results.

## 2. Materials and Methods

The Clear Castor Oil (CO) was bought from Jayant Agro-Organics Ltd. in Mumbai, India, with a hydroxyl value of 160 mg KOH/g, and an acid value of 1.6 mg KOH/g, a peroxide value of 5.0, a saponification value of 178, and a refractive index of 1.476. This study used Voranol 3010, a triolpolyether polyol from Jayant Agro-Organics Ltd., Mumbai, India. This study used tin (II) 2-ethylhexanoate from (Sigma-Aldrich, Bangalore, India) as a catalyst aided the foaming process. The diisocyanate used in this study was pTDI (polymeric toluene diisocyanate), bought from BASF under Lupranate 180. The catalyst for the foaming process was Niax B11 plus, while the surfactant for foam preparation was Niax L618 (Manali Petrochemicals Limited, Chennai, India). Distilled water was taken from the lab and used to create more foam, as it does not contain magnesium and calcium ions. These ions decrease the foam-forming capacity. The chemical composition of the components mentioned above is described in Table 1. Commercially available ZrO_2_ with an average size of 20–50 nm, BC nanoparticles with an average size of 20–50 nm, and GNP with an average size of 5–10 nm (supplied by Sigma Aldrich, Bangalore, India, 99.9% purity) were used as reinforcing materials. GNP, ZrO_2_, and BC are described in detail in Table 2.

**Figure 1:**
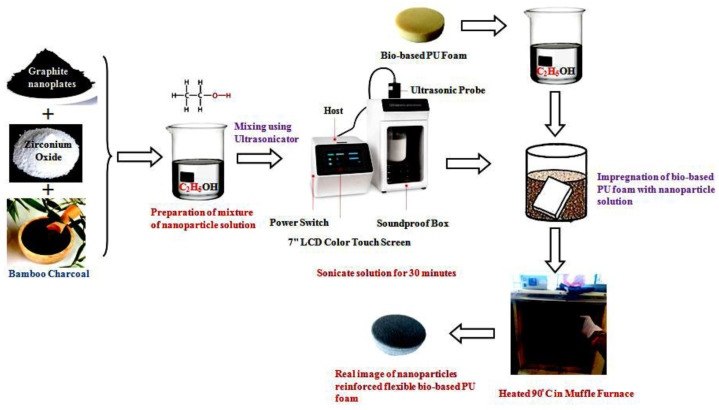
Experimental flow chart of nanoparticle reinforcement in flexible bio-based polyurethane foam.

Voranol 3010, a triol polyether polyol, and CO were dehydrated individually in a revolving evaporator below a vacuum at 110 °C for 24 h to remove moisture before the reaction, while nanoparticles were dried at 110 °C for 2 h. Then, in a mold with dimensions of 12 × 12 × 2 cm, pre-weighted (50 percent) polyether polyol and castor oil were combined. As a catalyst, tin (II) 2-ethylhexanoate was added to this mixture and mixed well for 5 h at 3600 r/min in an inert atmosphere in an oil bath at 65 °C. Before adding a determined quantity of amine catalyst and surfactant, the mixture was stored in an oven for 12 h at 60 °C. Distilled water was added to the mix and swirled for 18 s at 3500 rpm. Finally, premeasured pTDI was added to the mixture, and mixing continued for an additional 18 s at 2500 r/min. The viscous liquid was rapidly and carefully placed into an equipped mold and allowed to rise freely [51]. In this work, we have adopted the absorption and hydrothermal reduction methods for fabricating nanoparticle-reinforced flexible bio-based PU foam. The polyurethane foam was immersed entirely in an ethanol container at room temperature for about 10 min. For homogeneous distribution and to avoid nanoparticles settling at the bottom of the container, the necessary weight % of GNP, ZrO_2_, and BC was blended with ethanol in another container for 20 min using an ultrasonic probe sonicator. The polyurethane foam was then immersed completely in the nanoparticle solution for 20 min. The self-assembling nature of nanoparticles results in a consistent dispersion of nanoparticles throughout the foam. The soaked foam was heated in a furnace at 80 °C for 60 min to evaporate the ethanol. Once the foam was dried and was free of ethanol, it was removed from the furnace. The rectangular structure of size 12 × 12 × 2 cm was designed and fabricated in order to achieve a uniform symmetrical outer surface area. Due to this, the maximum amount of EM waves from the transmitting antenna is in contact with the material. The flow chart for making flexible bio-based PU foam is shown in Figure 1.

**Figure 2:**
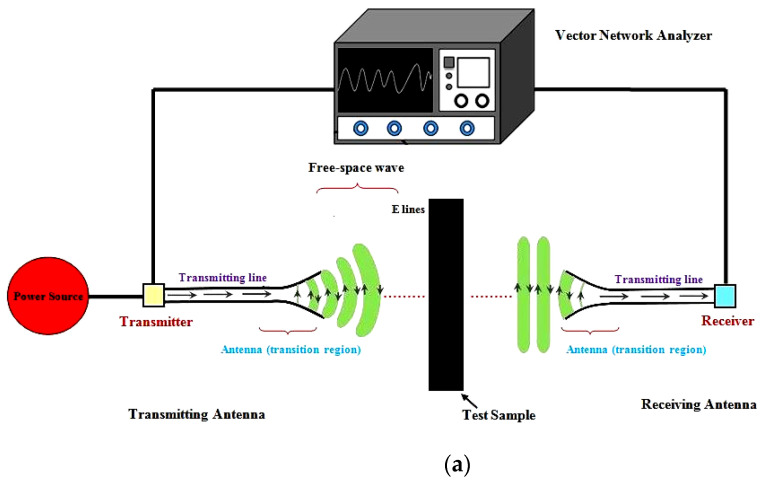

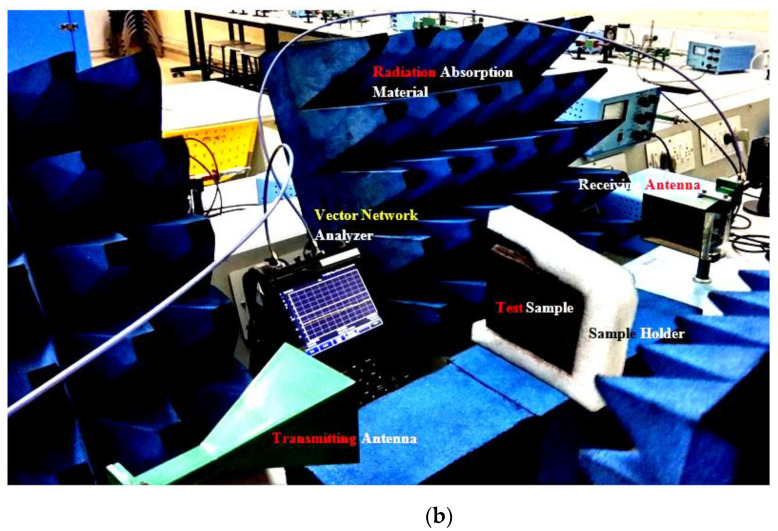
(**a**) Schematic diagram of EMI test setup. (**b**) Experimental setup for the Electromagnetic Interference test.

The electronic assembly’s actual shielding effectiveness is assessed using the open field or free space method. The radiated emissions that run away from the completed product are measured using this method. The EMI SE testing parameters include a typical network analyzer with model number N5230A PNA-L. It has a dynamic range of 108 dB and operates from 10 MHz–50 GHz. Waveguide measurements and OSL/TLL calibration are also available. The operational voltages for the experiments were +5 V and 15 V. Specimens were examined in the transmitter range at a frequency range of 8–12 GHz with a phase resolution of 1 degree. The length of the transmitter and receiving antennas is half a wavelength, with the feed line split precisely in the middle. The schematic illustration of the electromagnetic interference test setup is shown in Figure 2a. When the distance between the transmission source and the receiver exceeds k/2p, it is considered to be in the far-field protective district (where k is the frequency of the source and p is the 240 VAC power supply). The specimen was positioned in the center, 60 cm from the receiver and transmitter. The transmitter and receiver are linked to a vector network analyzer, which provides a plot of the EMI shielding effectiveness of the sample (see Figure 2b).

Response surface methodology examines the relationships between many explanatory variables and one or more response variables. The primary principle of RSM is to obtain an optimal response through a series of carefully prepared tests. Statistical methodologies such as RSM can optimize operational parameters to maximize the production of a specific material. In contrast to traditional methods, statistical techniques can be used to determine the interaction between process factors. If major explanatory factors are suspected, a more elaborate design, such as a central composite design (CCD), can be used to estimate a second-degree polynomial model; however, this is still only an approximation at best. Conversely, the second-degree model can be used to optimize the response variables of interest (maximize, minimize, or achieve a specific aim). Simulation software such as COMSOL Multiphysics is generally used in engineering, manufacturing departments, test laboratories, and scientific research to model designs, devices, and processes. Comsol Multiphysics with add-on modules provides specialized functionality for mechanical, fluid dynamics, electromagnetic interference, acoustics, and chemical applications. In our research, we use a computer-aided design (CAD) kernel to import models into the geometry section in a suitable format. A 3D periodic cartesian lattice with a unit cell containing arbitrarily shaped air-filled pores inside material with pre-defined frequency-dependent electromagnetic parameters is used to model the geometry. The radio frequency (RF) module in COMSOL Multiphysics is used to perform total field calculations of a TE or TM incident wave that excites the structure from the top at a predefined angle of incidence (Ɵ) (Port 1). Mesh refinement ensures accurate results by maintaining a minimum element quality of greater than 0.2. S_11_ and S_12_ of the structure are calculated over the X-Band with a sampling interval of 0.1 GHz. To prevent secondary reflections, the top of the cell is terminated by a perfectly matched layer (PML), and the bottom of the cell is removed by either a perfect electrically conducting (PEC) or a second port for the study of non-PEC-backed composites [52].

## 3. Results and Discussion

### 3.1. Field Emission Scanning Electron Microscope (FESEM)

**Figure 3:**
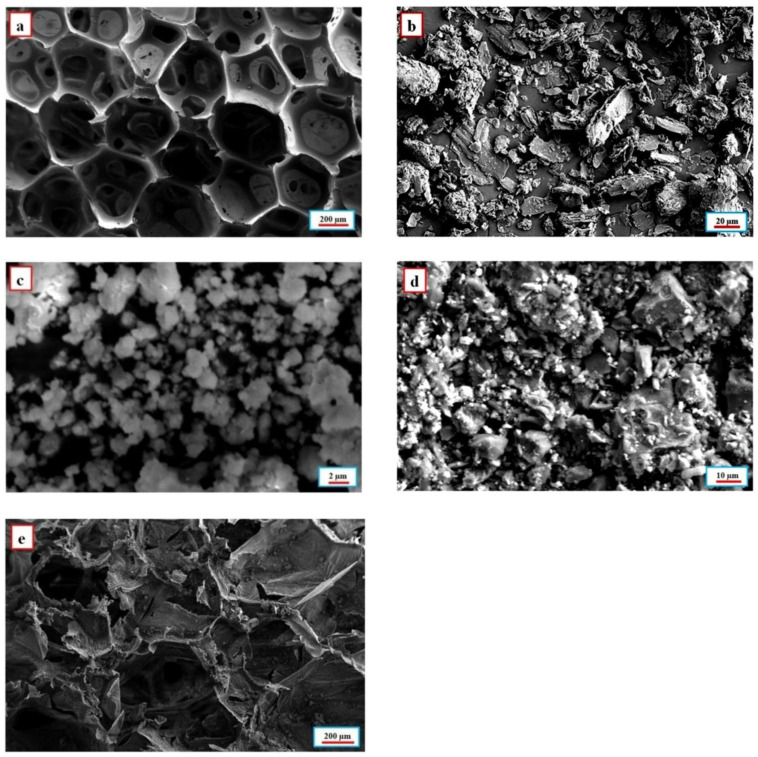
FESEM characterization of (**a**) Open cellular pores of bio-based flexible polyurethane foam, (**b**) Graphite nanoplates, (**c**) Zirconium oxide nanoparticles, (**d**) Bamboo charcoal nanoparticles, (**e**) Nanoparticle-reinforced flexible bio-based polyurethane foam.

The morphology of the nanoparticle-reinforced flexible bio-based polyurethane foam is examined by a field emission scanning electron microscope, as shown in Figure 3. Before being scanned, the specimens are gold-coated. Figure 3a shows the presence of an open cellular porous honeycomb structure morphology with an average pore size of 300 µm, and the thickness of the pore wall is 15 µm in fabricated bio-based polyurethane foam. It has been observed that there are plenty of nanoparticles surrounded by honeycomb pores. The flake-like structure in Figure 3b quickly reveals the presence of GNP nanoparticles. Under high GNP content, GNP overlapped each other to form an integral three-dimensional structure, which helps to improve the electromagnetic properties of the reinforced PU foam. From Figure 3c, it was speculated that GNP played a role in refining crystal grains, and promoting the aggregation and fusion of fine ZrO_2_. Thus, the spherical shape found in the FESEM denotes the existence of ZrO_2_. The accumulation of a large number of tiny particles confirms the presence of BC, as shown in Figure 3d. The uniformity across the sample is seen in Figure 3e due to the self-assembling characteristic of nanoparticles.

### 3.2. Fourier-Transform Infrared Spectroscopy (FTIR) Characterization

**Figure 4:**
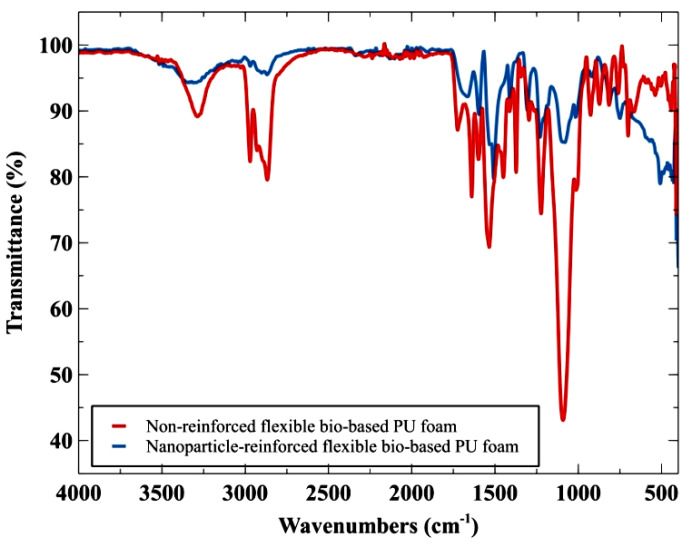
FTIR graph of non-reinforced and nanoparticle-reinforced flexible bio-based polyurethane foam.

The absorption, emission, and photoconductivity spectrum of solids, liquids, and gases are obtained using the Fourier-transform infrared spectroscopy (FTIR) technique. Between 4000 and 400 cm^−1^, the range is captured. The curves plotted between transmittance vs. wave number were used to identify the different functional groups in the various foams using a Nicolet NEXUS-670 FTIR instrument. Figure 4 compares the FTIR graph of non-reinforced flexible bio-based polyurethane foam (A0) and the best sample (A13), nanoparticle-reinforced flexible bio-based polyurethane foam. Here, the presence of carboxylic acid OH stretch is confirmed in non-reinforced foam around 3000–2800 cm^−1^ from stretching the peaks. N–H stretches of amines are present in the range of around 3300 cm^−1^ [53]. The asymmetric and symmetric bending vibrations of the HO–H produce peaks at 1648 cm^−1^ and 1381 cm^−1^. The carbonyl peak was found at 1724 cm^−1^, with the isocyanate peak (–NCO) disappearing at around 2270–2250 cm^−1^. There was no peak of hydroxyl at 3450 cm^−1^, indicating that all isocyanates contained in the pre-polymer constituents were fully used. While comparing the graph in Figure 4, the addition of nanoparticles to the bio-based polyurethane foam is readily seen. The medium-intensity sp2-hybridized C=C bonding in the 1680–1600 cm^−1^ range validates GNP. Three strong absorption bands are seen at wave numbers of 3380, 1650, and 550 cm^−1^ in tetragonal ZrO_2_ (Figure 4, blue line). The tension vibration of the hydroxyl groups on the ZrO_2_ surface produced two bands at 3380 and 1650 cm^−1^. The specimen contains other nanoparticles besides the GNP, as seen in the FTIR graph. The results are ambiguous, and there is a lot of noise due to the presence of alkanes and alkynes in the diverse compound mixture, indicating the presence of BC and GNP in the composite.

### 3.3. EMI SE Experimental Characterization

The sample was placed 30 cm from the transmitter and the receiver to keep the antenna in range. As shown in Figure 2b, the antenna and power supply were connected to a network analyzer, which provided S-parameter plots of the EMI SE of the specimen. The literature [31] and the following parameters, as stated in Equations (1)–(7), have been used to understand the experimental results of EMI SE:

Reflection Coefficient (R) = |S_11_|^2^; (1)

where S_11_ = Spectrum parameter in free air medium

Transmission Coefficient (T) = |S_12_|^2^; (2)

where S_12_ = Spectrum parameter of bio-based polyurethane foam

Absorption Coefficient (A) = 1 − R − T;(3)

Shielding Effectiveness Reflection (SE_ref_) = −10 log (1 − R) [dB] (4)

Shielding Effectiveness Absorption (SE_abs_) = −10 log (T/(1 − R)) [dB] (5)

Total Shielding Effectiveness (SE_total_) = (−)10 * log (T) [dB] or SE_ref_ + SE_abs_[dB] (6)

Error Percentage = (EMI SE_Actual_ − EMI SE_Predicted_)/(EMI SE_Actual_) [%] (7)

where

EMI SE_Actual_ = Actual Shielding Effectiveness of EMI from experimental analysis.

EMI SE_Predicted_ = Predicted Shielding Effectiveness of EMI from regression equation of statistical analysis.

**Figure 5:**
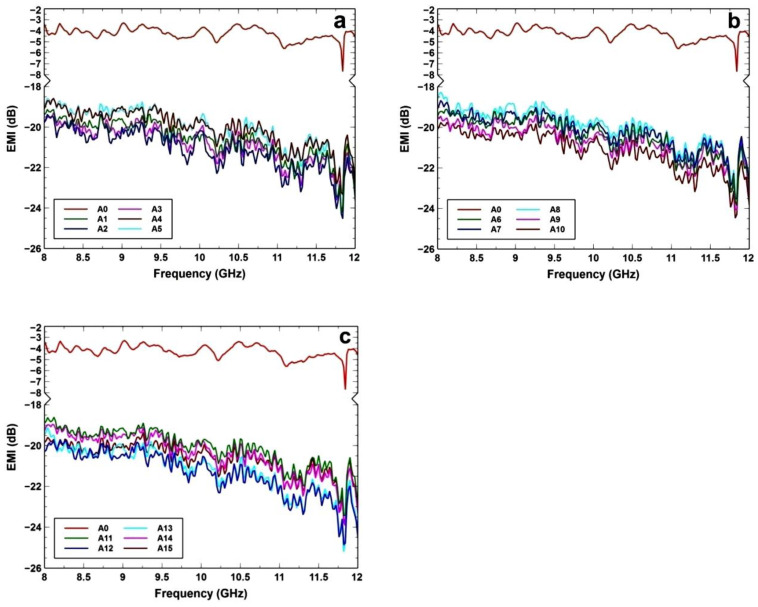
Evaluation of EMI values for (**a**) (A0, A1–A5), (**b**) (A0, A6–A10) and (**c**) (A0, A11–A15) of different flexible bio-based polyurethane foams with and without nanoparticle.

Figure 5 and Table 3 indicate the EMI data of different flexible bio-based polyurethane foams. Figure 5a (A0, A1–A5), Figure 5b (A0, A6–A10), and Figure 5c (A0, A11–A15) show the significant differences in EMI values of non-reinforced PU foam (A0) and nanoparticle-reinforced PU foam (A1–A15) in the frequency range 8–12 GHz. As shown in Figure 5, the specimen (A0) has a low electromagnetic interference value, ranging from 3.2 dB to 7.7 dB, because it transmits most of the EM radiation. Comparing the samples, in Figure 5a,b, the A2 and A10 samples have the highest EMI value, ranging from 19.2 dB to 24.5 dB. In Figure 5c, it is clear that sample A13 has a maximum electromagnetic interference value, ranging from 19.1 dB to 25.2 dB, which is the highest among all samples, implying that it absorbs the majority of the electromagnetic radiation, and so improves the EMI SE. It is evident from Figure 5 that the reinforced foams have a higher EMI value than the non-reinforced foam, as it shows a rise of roughly 18 dB over the flexible bio-based polyurethane foam without fillers.

Table 3 indicates the physical properties and EMI results for the experimental designs (L20) with different weight percentages from the RSM central composite design. The weight variation of each polyurethane foam indicates the presence of nanoparticles. The percentage of weight achieved after reinforcement using absorption and the hydrothermal process is shown in Table 3. As shown in Table 3, with a GNP weight percentage of 0.3, ZrO_2_ weight percentage of 0.3, and BC weight percentage of 2.5, specimen A13 exhibits the highest maximum EMI value of 25.2 dB. The EMI SE of nanocomposites is affected by morphology, GNP, ZrO_2_ and BC loading, incident radiation frequency, matrix composition, and nanocomposite thickness. Table 4 depicts the EMI shielding effectiveness, which is SE_Total_, of various nanocomposites over the frequency ranges of 8–12 GHz, derived using Equations(1)–(6), as well as the parameters obtained using Equations (1)–(4) for the free air medium shown in Table 5. Due to the limit of the measurement equipment, we only analyzed the effective contribution of different attenuating mechanisms in the X-band frequency range. Table 4 shows that in the frequency range of 8–12 GHz, the experimental specimen A13 has the highest EMI shielding effectiveness of −25.62 dB to −28.03 dB. Based on these findings, we concluded that the EMI SE of nanoparticle-reinforced PU foam has improved, comparing specimen A without nanoparticles, which has an EMI shielding effectiveness ranging from −8.66 dB to −17.75 dB. The shielding effectiveness value’s pessimistic sign denotes the breakdown of the electromagnetic wave power to the incident electromagnetic wave.

### 3.4. EMI SE Statistical Analysis

The weight percentage of GNP, ZrO_2_, and BC are the input parameters, while EMI Shielding Effectiveness is the output parameter. The DOE tool was an RSM model, the central composite design approach. Table 6 depicts the various levels of input parameters used. Using Minitab software, ANOVA was used to determine the effects of the weight percentage of GNP, ZrO_2_, and BC on the EMI shielding effectiveness of flexible bio-based polyurethane foam.

**Figure 6:**
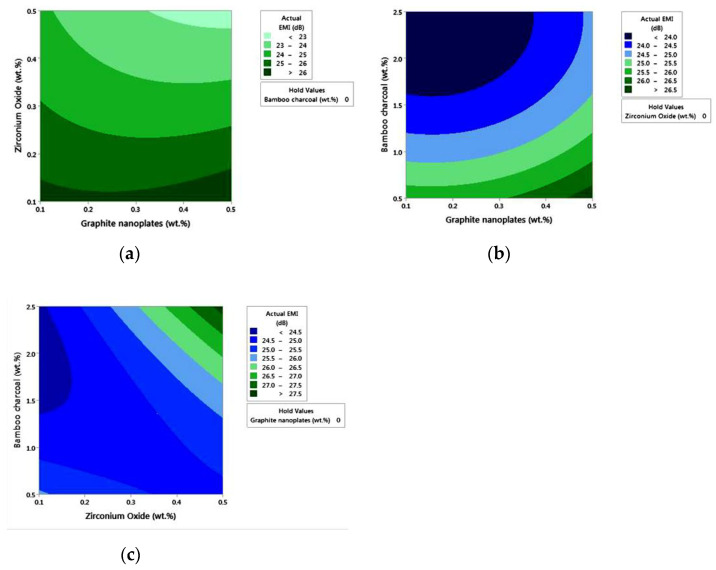
Contour plots displaying the impact of (**a**) GNP and ZrO_2_ on EMI effect, (**b**) GNP and BC on EMI effect, (**c**) BC and ZrO_2_ on EMI effect.

Table 7 represents the ANOVA findings, and the *p*-value is 0.6, indicating that all the data and the model are statistically significant and fit the experimental results. As shown by the findings, the model may be used to predict the results with a 95% confidence level. In Figure 6, the contour plots indicate the interaction between the input parameters and output response. Figure 6a shows a maximum EMI value greater than 26 dB for the composition of GNP with a weight of 0.1–0.5 percent and ZrO_2_ with a weight of 0.1–0.17. Figure 6b shows a maximum EMI value greater than 26.5 dB for the composition of GNP with a weight of 0.45–0.5 percent and BC with a weight of 0.5–0.6 percent. Finally, Figure 6c shows a maximum EMI value greater than 27.5 dB for the composition of BC with a weight of 2.3–2.5 percent and ZrO2 with a weight of 0.45–0.5 percent. The regression equation produced using ANOVA is Equation (8). In Equation(8), A, B, and C represent the weight percent of GNP, ZrO_2_, and BC, respectively. It forecasts EMI values by substituting the corresponding weight percent from Table 6. Table 8 compares the actual EMI values acquired through the experimental investigation with the expected EMI values obtained from Equation (8). The Electromagnetic Interference value has been employed for numerical analysis, since it is directly related to the EMI SE. Table 8 demonstrates that the highest error % between the real and statistically predicted electromagnetic interference is less than 5%, demonstrating the regression equation’s validity.

EMI = 27.29 − 2.7 ∗ A − 7.1 ∗ B − 2.89 ∗ C + 8.6 ∗ A ∗ A + 4.9 ∗ B ∗ B + 0.595 ∗ C ∗ C − 12.5 ∗ A ∗ B + 0.00 ∗ A ∗ C + 5.00 ∗ B ∗ C(8)

**Figure 7:**
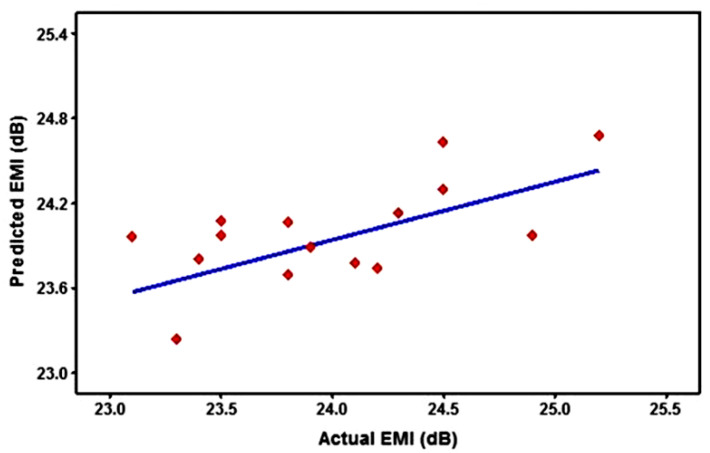
Actual vs. Predicted Electromagnetic Interference value.

A scatter plot (Figure 7) is a graphic or mathematical diagram that uses Cartesian coordinates to display values for two variables for data collection. Figure 7 indicates the contrast between the real value on the X-axis and projected EMI values on the Y-axis. As seen in Figure 7, there is a linear connection between the expected and real values of EMI. This underpins the significance of the regression equation derived from the analysis of variance in determining the best range of various particles to utilize for reinforcement to get the highest EMI.

**Figure 8:**
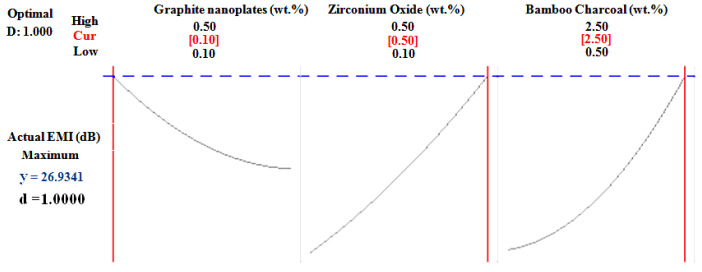
Optimization curvature of factor for different nanoparticles influencing EMI value.

Table 9 and Figure 8 represent the optimal weight percent of GNP, ZrO_2_, and BC for the best EMI value through the RSM optimization study. It shows that the combination of 0.1 weight percent GNP, 0.5 weight percent ZrO_2_, and 2.5 weight percent BC provides the highest EMI SE data, i.e., 28.61 dB, with a desirability value of 1.

Table 10 compares EMI SE with different materials, dominant mechanisms, frequency range, and thickness. As shown in Table 10, this work presents a relatively lightweight polyurethane foam that can improve EMI SE by adding nanoparticles.

### 3.5. EMI SE Computational Analysis

The numerical study of the EMI shielding effect on polyurethane foam was carried out by RF module in COMSOL Multiphysics^®^. The high-frequency waves used in this are formulated by Maxwell–Ampere’s and Faraday’s laws,

∇*xH* = *J* + *∂D*/*∂t* (9)

∇*xE* = −*∂B*/*∂t* (10)

where *J* is the current density, *E* is the electric field intensity, *D* is the electric flux density, *B* is the magnetic flux density, and *H* is the magnetic field intensity; where the governing wave equation for the electric field intensity *E* is the electromagnetic waves. The frequency domain interface can be written in the form,

(11) $$\nabla \times \mu_{r}^{-1}\;(\nabla \times E)-k_{0}^{2}n^{2}E=0$$

where *n*^2^ = $\left(\varepsilon_{r}-\frac{j\sigma }{\omega \varepsilon_{0}}\right)$, where n is the refractive index, and the wave number of free space k_0_ is defined as

**Figure 9:**
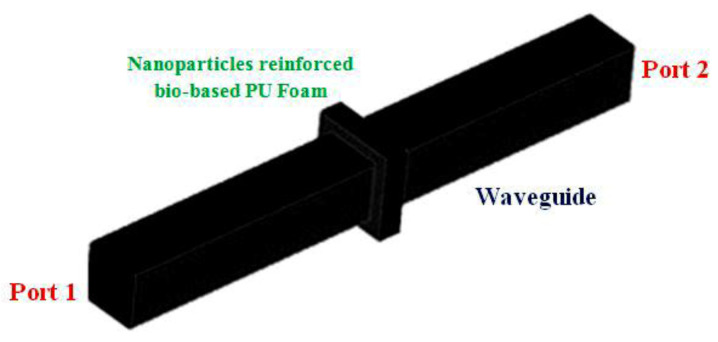
3-D model for analyzing electric field intensity.

*k*_0_ = *ω√ε*_0_*μ*_0_ = *ω*/*c*_0_ (12)

and the computation of wave through both antennas (transmitter and receiver) can be defined from the below equations,

(13) $$\mathrm{for}\;\mathrm{port}\;1,\;S=\frac{f_{\partial \Omega }\left(E-E_{1}\right).E_{1}}{f_{\partial \Omega }E_{1}.E_{1}}$$

(14) $$\mathrm{for}\;\mathrm{port}\;2,\;S=\frac{f_{\partial \Omega }E.E_{2}}{f_{\partial \Omega }E_{2}.E_{2}}$$

where *ω* is the angular frequency, *σ* is the electrical conductivity, *S* is the scattering parameter, $f_{\partial \Omega }$ is the frequency domain, *ε*_0_ and *µ*_0_ are the free space permittivity and permeability, and *ε_r_* and *µ_r_* are the relative permittivity and permeability, respectively, and *c*_0_ is the speed of light in vacuum [45]. The simplified geometry of flexible bio-based polyurethane foam of size 12 × 12 × 2 cm was imported in COMSOL Multiphysics using CAD import module capabilities. A unit cell was then built around the imported geometry applying Floquet-periodic boundary conditions on its four sides, creating an infinite 3D array of simplified cellular structures that act like the original model of the bio-based polyurethane foam. Figure 9 shows one port on the bottom face and another on the upper faces of the unit cell to reassemble the experimental conditions described in the Methodology section. Ports 1 and 2 emulate the transmitter and the receiver antenna, respectively.

**Figure 10:**
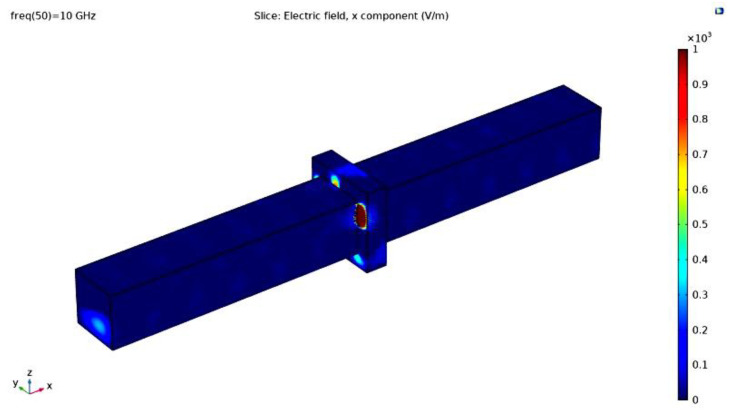
Simulation of Electric Field Intensity through COMSOL Multiphysics.

**Figure 11:**
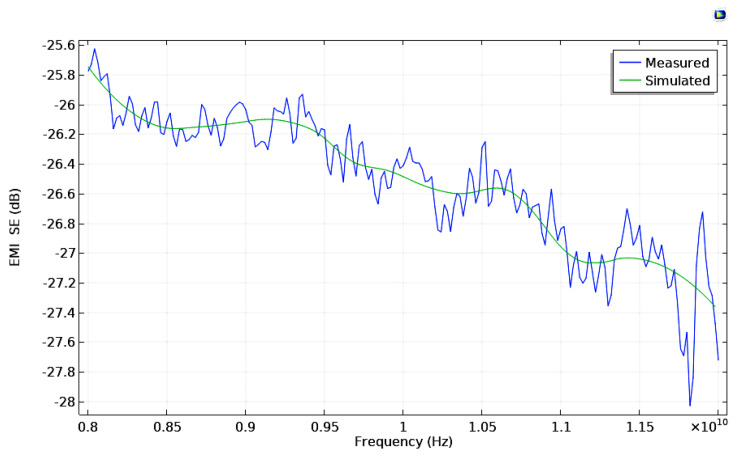
Shielding Effectiveness comparison between measured and simulated sample.

The distance between ports was 42 cm. The imported simplified foam model was placed between Port 1 and Port 2. The electric mode field amplitude in Port 1 was configured following the relation, and their values are permittivity = 1.126 × 10^−11^ F/m, permeability = 1.257 × 10^−6^ H/m, and electrical conductivity = 3.588 × 10^−14^ S/m, where the waveguide width parameter is equal to 2.5 cm. The elevation angle of incidence is 0, which means the electric field is normal to the geometry surface. The mathematical equation for component y of the electric mode field amplitude matches the spatial propagation behavior exhibited by a classic pyramidal horn antenna. The material chosen for the polymeric foam was (built-in) polyurethane, and the domain outside and inside (the bubbles of) the cellular structure was filled with air. The electric and magnetic material properties are required to solve the governing equations of the mathematical model. The frequencies analyzed (parametric sweep) went from 8 GHz to 12 GHz, with a 500 MHz interval between computed values. The norm of the electric field and power flow at 10 GHz are shown in Figure 10, respectively. Finally, the shielding effectiveness values were computed for each frequency analyzed for the physical model-simulated and the flexible bio-based PU foam. These scenarios are summarized in frequency spectra, as shown in Figure 11. The results carry a relative error of 2.48% between the EMI SE of the manufactured material, which peaked at 28.03 dB, while the maximum value reached for the simulated model is near 27.35 dB.

## 4. Conclusions

Nanoparticle-reinforced flexible bio-based PU foam samples were easily produced using absorption and hydrothermal reduction reaction methods, and they showed to be dependable and delicate with precise control. In total, 15 samples were made using different compositions determined by an RSM central composite design technique. Sample A13 (0.3 wt.%, 0.3 wt.%, and 2.5 wt.% for GNP, ZrO_2_, and BC, respectively) depicts a maximum EMI SE of 28.03 dB in the 8–12 GHz frequency band, which is improved compared to regular PU foam without reinforcement. The presence and uniformity of nanoparticles in the PU foam have been authenticated through FESEM and FTIR. Influential parameters have been discovered for statistical computation, and their amounts have been set using ANOVA. The regression equation was persistent, and the EMI SE was obtained using the iterative results of the different samples and the ANOVA for EMI. The difference between the regression equation predicted EMI values and those measured in the experiment are less than 5%. The ideal weight percentage for attaining the highest EMI SE value of 28.61 dB is the combination of 0.1 wt.%, 0.5 wt.%, and 2.5 wt.% for GNP, ZrO_2_, and BC, respectively, through the optimization curve.

The electromagnetic behavior of a geometrically simplified open-cell flexible bio-based polyurethane foam was simulated using the RF module in COMSOL Multiphysics. The results carry a relative error of 2.48% between the EMI SE of the manufactured material, which peaked at 28.03 dB, while the maximum value reached for the simulated model is near 27.35 dB. For specific frequency values, the experimental and simulated results indicate that the sample A13 material can act as a resonance cavity, considerably attenuating the incoming EM radiation. The nanocomposite flexible bio-based PU foam is potentially biodegradable, lightweight, flexible, and low-cost. As a result, it can be utilized as a long-term replacement for traditional PU foam in shielding applications, such as terrestrial data transfer, radio operations, satellite communication body part covering, and radio navigation and positioning. The future scope of this work can be extended with the reinforcement of different nanoparticles for enhancing the EMI shielding.

## Figures and Tables

**Table 1:** Chemical composition of CO-based flexible polyurethane foam.

Components	Weight of Components in g
Voranol 3010	50
Castor Oil (CO)	50
pTDI	46.2
Surfactant	3.6
Tin Catalyst	1.0
Amine Catalyst	0.25
Distilled Water	0.9

**Table 2:** Details of nanoparticles reinforced in CO-based flexible polyurethane foam.

Nanoparticles	Density (g/cm^3^)	Melting Point (°C)	Particle Size (nm)
Graphite nanoplates (GNP)	2.267	3650	5–10
Zirconium Oxide (ZrO_2_)	5.680	2715	20–50
Bamboo Charcoal (BC)	0.690	1200	20–50

**Table 3:** Physical properties and EMI results for the experimental design (L20).

Specimen	Weight of PU Foam without Reinforcement (g)	Graphite Nanoplates (wt.%)	Zirconium Oxide (wt.%)	Bamboo Charcoal (wt.%)	Weight of PU Foam with Reinforcement		Weight Achieved after Reinforcement (%)	Density (g/cm^3^)	EMI (dB)
					Theoretical Value (g)	Actual Value (g)			
A0	4.217	-	-	-	-	-	-	0.015	7.71
A1	4.137	0.4	0.4	2	15.337	16.128	94.84	0.056	24.3
A2	4.281	0.2	0.4	2	14.681	15.552	94.07	0.054	24.5
A3	4.358	0.2	0.4	1	10.758	12.069	87.81	0.042	24.2
A4	4.330	0.4	0.4	1	11.530	12.960	87.60	0.045	23.3
A5	4.284	0.4	0.2	2	14.684	15.552	94.09	0.054	23.1
A6	4.343	0.4	0.2	1	10.743	11.808	90.09	0.041	23.8
A7	4.305	0.2	0.2	1	9.905	11.369	85.22	0.040	23.5
A8	4.325	0.2	0.2	2	13.925	15.264	90.38	0.053	23.5
A9	4.116	0.5	0.3	1.5	13.316	14.688	89.70	0.051	24.1
A10	4.260	0.1	0.3	1.5	11.860	12.384	95.58	0.043	24.5
A11	4.366	0.3	0.5	1.5	13.566	14.400	93.85	0.050	23.4
A12	4.304	0.3	0.1	1.5	11.904	12.960	91.13	0.045	24.9
A13	4.353	0.3	0.3	2.5	16.753	16.953	98.81	0.058	25.2
A14	4.365	0.3	0.3	0.5	8.765	10.025	85.62	0.035	23.9
A15	4.267	0.3	0.3	1.5	12.667	12.960	97.69	0.045	23.8
A16	4.267	0.3	0.3	1.5	12.667	12.960	97.69	0.045	23.8
A17	4.267	0.3	0.3	1.5	12.667	12.960	97.69	0.045	23.8
A18	4.267	0.3	0.3	1.5	12.667	12.960	97.69	0.045	23.8
A19	4.267	0.3	0.3	1.5	12.667	12.960	97.69	0.045	23.8
A20	4.267	0.3	0.3	1.5	12.667	12.960	97.69	0.045	23.8

**Table 4:** Diagnostic calculation of EMI SE for non-reinforced and reinforced samples.

Specimen	Transmission Coefficient (T) (dB)	Absorption Coefficient (A) (dB)	Shielding Effectiveness Absorption (SE_abs_) (dB)	Total Electromagnetic Interference Shielding Effectiveness (EMI SE_Total_) (dB)
A0	7.34 ~ 59.57	−9.07 ~ −78.82	−6.28 ~ −4.91	−8.66 ~ −17.75
A1	364.81 ~ 590.49	−366.54 ~ −609.74	−23.24 ~ −14.87	−25.62 ~ −27.71
A2	372.49 ~ 600.25	−374.22 ~ −619.5	−23.33 ~ −14.94	−25.71 ~ −27.78
A3	372.49 ~ 585.64	−374.22 ~ −604.89	−23.33 ~ −14.83	−25.71 ~ −27.68
A4	345.96 ~ 542.89	−347.69 ~ −562.14	−23.01 ~ −14.50	−25.39 ~ −27.35
A5	345.96 ~ 533.61	−347.69 ~ −552.86	−23.01 ~ −14.43	−25.39 ~ −27.27
A6	364.81 ~ 566.44	−366.54 ~ −585.69	−23.24 ~ −14.69	−25.62 ~ −27.53
A7	349.69 ~ 552.25	−351.42 ~ −571.5	−23.06 ~ −14.58	−25.44 ~ −27.42
A8	334.89 ~ 552.25	−336.62 ~ −571.5	−22.87 ~ −14.58	−25.25 ~ −27.42
A9	376.36 ~ 580.81	−378.09 ~ −600.06	−23.38 ~ −14.80	−25.76 ~ −27.64
A10	392.04 ~ 600.25	−393.77 ~ −619.5	−23.55 ~ −14.94	−25.93 ~ −27.78
A11	345.96 ~ 547.56	−347.69 ~ −566.81	−23.01 ~ −14.54	−25.39 ~ −27.38
A12	388.09 ~ 620.01	−389.82 ~ −639.26	−23.51 ~ −15.08	−25.89 ~ −27.92
A13	364.81 ~ 635.04	−366.54 ~ −654.29	−23.24 ~ −15.18	−25.62 ~ −28.03
A14	357.21 ~ 571.21	−358.94 ~ −590.46	−23.15 ~ −14.72	−25.53 ~ −27.57
A15	372.49 ~ 566.44	−374.22 ~ −585.69	−23.33 ~ −14.69	−25.71 ~ −27.53
A16	372.49 ~ 566.44	−374.22 ~ −585.69	−23.33 ~ −14.69	−25.71 ~ −27.53
A17	372.49 ~ 566.44	−374.22 ~ −585.69	−23.33 ~ −14.69	−25.71 ~ −27.53
A18	372.49 ~ 566.44	−374.22 ~ −585.69	−23.33 ~ −14.69	−25.71 ~ −27.53
A19	372.49 ~ 566.44	−374.22 ~ −585.69	−23.33 ~ −14.69	−25.71 ~ −27.53
A20	372.49 ~ 566.44	−374.22 ~ −585.69	−23.33 ~ −14.69	−25.71 ~ −27.53

**Table 5:** Diagnostic calculation of Reflection Coefficient (R) and Shielding Effectiveness Reflection (SE_ref_) from Equations (1) and (4).

Specimen	Reflection Coefficient (R) (dB)	SE Reflection (SE_ref_) (dB)
Free Air Medium	2.73 ~ 20.25	−2.38 ~ −12.84

**Table 6:** Different levels of nanoparticles worn in flexible bio-based polyurethane foam.

Code	Parameters	Levels				
		−2	−1	0	1	2
A	Graphite nanoplates (wt.%)	0.1	0.2	0.3	0.4	0.5
B	Zirconium Oxide (wt.%)	0.1	0.2	0.3	0.4	0.5
C	Bamboo Charcoal (wt.%)	0.5	1	1.5	2	2.5

**Table 7:** ANOVA for electromagnetic interference (dB).

Source	DF	Sum of Squares	Mean Square	F-Value	*p*-Value
Model	9	2.17432	0.241591	0.78	0.644
Linear	3	0.59970	0.199900	0.64	0.605
A	1	0.01921	0.019208	0.06	0.809
B	1	0.13300	0.132998	0.43	0.528
C	1	0.55625	0.556249	1.79	0.211
Square	3	0.63682	0.212273	0.68	0.583
A * A	1	0.18753	0.187532	0.60	0.456
B * B	1	0.06003	0.060032	0.19	0.670
C * C	1	0.55718	0.557175	1.79	0.211
2-Way Interaction	3	0.62500	0.208333	0.67	0.590
A * B	1	0.12500	0.125000	0.40	0.541
A * C	1	0.00000	0.000000	0.00	1.000
B * C	1	0.50000	0.500000	1.60	0.234
Error	10	3.11568	0.311568		
Lack-of-Fit	5	3.11568	0.623136	*	*
Pure Error	5	0.00000	0.000000		
Total	19	5.29000			

**Table 8:** EMI error estimation between real and statistically anticipated values.

Specimen	Real Value of EMI (dB)	Statistical Value of EMI (dB)	Residual	Error%
A1	24.3	24.1	0.2	0.69
A2	24.5	24.6	−0.1	−0.56
A3	24.2	23.7	0.5	1.88
A4	23.3	23.2	0.1	0.27
A5	23.1	23.9	−0.8	−3.73
A6	23.8	24.0	−0.2	−1.12
A7	23.5	24.0	−0.5	−2.44
A8	23.5	23.9	−0.4	−2.00
A9	24.1	23.7	0.4	1.32
A10	24.5	24.2	0.3	0.83
A11	23.4	23.8	−0.4	−1.74
A12	24.9	23.9	1.0	3.72
A13	25.2	24.6	0.6	2.04
A14	23.9	23.8	0.1	0.02
A15	23.8	23.6	0.2	0.44
A16	23.8	23.6	0.2	0.44
A17	23.8	23.6	0.2	0.44
A18	23.8	23.6	0.2	0.44
A19	23.8	23.6	0.2	0.44
A20	23.8	23.6	0.2	0.44

**Table 9:** Optimum wt.% of graphite nanoplates, zirconium oxide and bamboo charcoal.

Solution	Graphite Nanoplates (wt.%)	Zirconium Oxide (wt.%)	Bamboo Charcoal (wt.%)	EMI SE FIT	Desirability
**1**	0.1	0.5	2.5	28.61	1

**Table 10:** Performance of EMI SE based on materials, dominant mechanism, frequency range, and thickness.

Matrix and Fillers Used	Dominant Mechanism	Frequency Range (GHz)	Thickness (mm)	EMI SE (dB)	Ref.
PU Foam/0.75 wt.% MWCNT/1.5 wt.% CuO/1.5 wt.% BC	Absorption	8–12	20	22.6	[31]
PU Foam/30 wt.% Gr	Absorption	8–12	5	44	[54]
Epoxy/1 wt.% MWCNT/5.5 wt.% BC/5.5 wt.% CuO	Absorption	8–12	3	24.52	[55]
PU Foam/18.7 wt.% GO	Absorption	8–12	9	30–35	[56]
Epoxy/Graphene	Absorption	8.2–12.4	0.8 × 10^−6^–1.1 × 10^−6^	21	[57]
Fe_3_O_4_/GN	Reflection	8–12	0.2–0.25	21–24	[58]
Fe_3_O_4_/RGO/PEI	Absorption	8–12	2.5	14–18	[59]
MWCNT/PC	Absorption	8–12	1.85	25	[60]
RGO films	Reflection	8–12	0.0084	18–22	[61]
CVD graphene/PDMS	Absorption	8–12	1	20	[62]
PU/0.3 wt.% GNP/0.3 wt.% ZrO_2_/2.5 wt.% BC	Absorption	8–12	20	28.03	Current research

## Data Availability

Not applicable.

## Nomenclature

EMI SE	Electromagnetic Interference Shielding Effectiveness
RSM	Response Surface Methodology
CCD	Central Composite Design
FESEM	Field Emission Scanning Electron Microscope
FTIR	Fourier-transform infrared spectroscopy
GNP	Graphite nanoplates
ZrO_2_	Zirconium Oxide
BC	Bamboo Charcoal
wt.%	Weight Percentage
Eqs	Equation
CAD	Computer-Aided Design
RF	Radio Frequency
